# Novel Methodological Tools for Behavioral Interventions: The Case of HRV-Biofeedback. Sham Control and Quantitative Physiology-Based Assessment of Training Quality and Fidelity

**DOI:** 10.3390/s21113670

**Published:** 2021-05-25

**Authors:** Ewa Ratajczak, Marcin Hajnowski, Mateusz Stawicki, Włodzisław Duch

**Affiliations:** 1 Institute of Psychology, Faculty of Philosophy and Social Sciences, Nicolaus Copernicus University in Toruń, 87-100 Toruń, Poland; 2 Department of Informatics, Faculty of Physics, Astronomy and Informatics, Nicolaus Copernicus University in Toruń, 87-100 Toruń, Poland; wduch@umk.pl; 3 Centre for Modern Interdisciplinary Technologies, Nicolaus Copernicus University in Toruń, Wileńska 4, 87-100 Toruń, Poland; matestawicki@gmail.com; 4 Institute of Information and Communication Research, Faculty of Philosophy and Social Sciences, Nicolaus Copernicus University in Toruń, 87-100 Toruń, Poland; 267603@stud.umk.pl

**Keywords:** psychophysiology, heart rate variability biofeedback, training quality, training fidelity, sham training control

## Abstract

Scientific research on heart rate variability (HRV) biofeedback is burdened by certain methodological issues, such as lack of consistent training quality and fidelity assessment or control conditions that would mimic the intervention. In the present study, a novel sham HRV-biofeedback training was proposed as a credible control condition, indistinguishable from the real training. The Yield Efficiency of Training Index (YETI), a quantitative measure based on the spectral distribution of heart rate during training, was suggested for training quality assessment. A training fidelity criterion derived from a two-step classification process based on the average YETI index and its standard deviation (YETI_SD_) was suggested. We divided 57 young, healthy volunteers into two groups, each subjected to 20 sessions of either real or sham HRV-biofeedback. Five standard HRV measures (standard deviation of the NN (SDNN), root mean square of the standard deviation of the NN (RMSSD), total power, low-frequency (LF), and high-frequency (HF) power) collected at baseline, after 10 and 20 sessions were subjected to analysis of variance. Application of a training fidelity criterion improved sample homogeneity, resulting in a substantial gain in effect sizes of the group and training interactions for all considered HRV indices. Application of methodological amendments, including proper control conditions (such as sham training) and quantitative assessment of training quality and fidelity, substantially improves the analysis of training effects. Although presented on the example of HRV-biofeedback, this approach should similarly benefit other behavioral training procedures that interact with any of the many psychophysiological mechanisms in the human body.

## 1. Introduction

### 1.1. Heart Rate Variability (HRV)-Biofeedback 

The links between general well-being and heart rate variability (HRV), defined as the variability of time intervals between consecutive heartbeats, have been well-documented in the scientific literature (for review, see: [1,2]). HRV is considered to be a robust and reliable indicator of the general condition of the cardiovascular system. It serves as an essential biomarker of physiological changes related to baroreflex and cardiac vagal activity. Low variability of the heart rate suggests deficient vagal influence [3]. Moreover, several central nervous system (CNS) areas involved in the perception of threat and safety (including the amygdala and the medial prefrontal cortex) are also associated with HRV control. Therefore, HRV parameters may index the degree of integration of perceptual, motor, interoceptive, and memory systems into one super-system, a gestalt representation of situations and likely adaptive responses. These findings explain how HRV may reflect essential bodily functions associated with adaptability and health [4]. Within such a framework, HRV indices may be viewed not only as indicators of cardiological health but also psychophysiological markers of general well-being. Indeed, high values of HRV have been reported to represent psychological resilience and ability to cope with stress, while low HRV has been associated with poor mental and somatic health [5].

Several factors increase HRV, such as positive mood and emotions [6], moderate levels of physical exercise [7], healthy diet [8], humor [9], music [10], and meditation [11,12]. It is possible to voluntarily increase HRV with the biofeedback technique in which biosignals from the body are collected by a sensor and presented back to the trainee in a visual or auditory form (usually visualized on a computer screen). During HRV-biofeedback training, the pulse is assessed based on electrocardiography (ECG) or pulse plethysmography (PPG) measurements, and variability of the heart rate (HR) is displayed as online feedback. Presentation of the biofeedback signal allows for voluntary modification of HRV fluctuations via breathing due to the respiratory sinus arrhythmia (RSA) phenomenon. The RSA causes the heart rate to increase upon inhalation and decrease during exhalation. In-phase synchronization of the respiratory HR fluctuations with the oscillations caused by the sympathovagal control of heart rate and blood pressure results in a state of cardio-vascular resonance. This state, also known as ‘coherence’, is characterized by maximization of HRV [5]. Although individual resonance frequencies of breathing differ slightly, the population average approximates 0.1 Hz, i.e., six breaths per minute [2].

The HRV-biofeedback training has been used in addition to psycho- and pharmacotherapy, as well as a stand-alone alternative to more traditional types of treatment [13]. It has been classified as an effective treatment for several disorders, with the best results achieved for stress and anxiety [14,15]. Numerous papers have reported successful HRV-biofeedback intervention in the case of a whole variety of somatic diseases and mental disorders, as well as beneficial applications of this technique for healthy participants, improving cognitive functioning and physical performance [16].

Several mechanisms have been proposed to underlie the regulatory effects of HRV-biofeedback, such as mechanical stretching of the airways, anti-inflammatory influences, relaxation, and meditative effects [2]. Recently, more complex mechanisms have been suggested that combine physiological and psychological effects with neurodynamics. HRV-biofeedback training affects brain functions at several levels of the CNS via bidirectional vagal connections between the heart and the brain. Stabilization of autonomic reactivity through baroreflex strengthening during training was proposed to translate directly on blood pressure modulation and indirectly on other autonomically and emotionally mediated reflexes [17]. Central plasticity is affected, leading to structural and functional changes, further reducing sympathetic arousal and the hypothalamic–pituitary–adrenal (HPA) axis activity [18,19]. Moreover, afferent information from the heart reaches higher brain regions, such as the central autonomic network, the limbic system, the reticular activating system, and the diffuse noradrenergic projection system [20]. Likewise, neuroimaging studies of the medial visceromotor network, particularly the prefrontal and the anterior cingulate cortex [21], point towards central effects. These structures are responsible for parasympathetic neurocardiac regulation based on reciprocal brain–heart interconnections, as well as being involved in the initiation and maintenance of adaptive control of emotions and goal-directed behavior [21]. Last but not least, a most recently suggested modus operandi was associated with changes in functional connectivity. This assumes that high-amplitude physiological fluctuations stimulate oscillatory activity within the brain, strengthening functional connectivity between affected regions [22].

### 1.2. Methodological Issues in HRV-Biofeedback Research 

The importance of following standard procedures in HRV-related research has been previously stressed in the literature. The Task Force of the European Society of Cardiology and the North American Society of Pacing and Electrophysiology laid grounds for standardized methodology, including nomenclature, definitions, methods of measurements, and interpretation of parameters [23]. Quintana and colleagues have recommended guidelines regarding the reporting and planning of HRV studies, such as participant selection, data collection, analysis and cleaning, and HRV calculation [24,25]. Shaffer and Ginsberg have provided an overview of various HRV metrics and stressed the influence of measurement context, including recording period length, subject age, and sex, on baseline HRV values. Laborde and coworkers have discussed methodological aspects related to HRV in psychophysiological research crucial for interpretation and cross-laboratory comparisons of findings. These recommendations and practical advice concern such issues as experimental designs, sample size, choice of HRV indices, confounding variables, standards for data collection, analysis, and reporting [26]. Moreover, a broad range of factors affecting HRV parameters by influencing cardiac vagal control has been identified and integrated into a unifying conceptual framework based on the neurovisceral integration model by Laborde and coauthors [27]. These are all important guidelines to follow while conducting research focused on HRV-biofeedback. 

On the other hand, despite a lot of research, various healthcare specialists still consider biofeedback an experimental, investigational, and largely unproven form of treatment. Official standards for research methodology in the field of biofeedback have existed since 2001, established by a Task Force commissioned by the Association for Applied Psychophysiology and Biofeedback (AAPB) and the International Society for Neuronal Regulation (ISNR) [3]. Nevertheless, biofeedback-related research still lacks the necessary methodological consistency. Studies of HRV-biofeedback are not an exception to this rule. Establishing a robust method to quantify training efficiency and fidelity and a reliable control condition would arguably provide the most benefit for the field [3].

Different control group designs can be found in the HRV-biofeedback literature, the most popular being no intervention or waiting list controls [28,29,30,31,32,33]. However, it is well known that expectations, attention, motivation, and involvement play key roles in achieving the effects of training. Passive control is much less cognitively demanding than biofeedback training and overlooks any potential impact of the expectations towards an intervention. Active control conditions, such as relaxation [34,35], neurofeedback [36,37], and placebo video clips [38,39] control this effect only partially. Certain active control conditions, including presentation of biofeedback without instructions concerning the pace of breathing or maximization of HRV [40], slow breathing [41], and concentrative passive feedback (HR displayed over 10-s intervals instead of real-time) [42], are more demanding, but still not optimal. Perfect control conditions should provide identical context as the intervention (external conditions, the attention and effort required from the trainee, the attention received from the trainer, etc.) while differing in as few details as possible. Ideally, the control condition does not introduce any treatment-specific processes within the body and hence produces no potential therapeutic effects. A condition closest to satisfying these requirements is the sham biofeedback approach, successfully applied in neurofeedback and other biofeedback modalities [43,44,45,46,47,48]. Sham biofeedback setup mimics the training conditions, with the only difference being the origin of the signal presented to the trainee. Instead of their own feedback, the sham trainee is shown a pre-recorded or random signal, which does not reflect their actual physiological state or ongoing processes. One specific drawback of this method includes the ethical implications of making the participants believe that they are being treated. Another potential problem concerns more perceptive trainees who may discover the ongoing deception, raising a requirement for sham biofeedback to be designed and administered very carefully.

To our best knowledge to date, sham biofeedback training in the HRV modality has been applied only once in a study investigating the effects of HRV-biofeedback in the treatment of major depression disorder [49]. The protocols for real and sham HRV-biofeedback training were almost identical. Trainees in both groups were presented with their own biofeedback signal and received the same instructions on the breathing technique and frequency of training sessions, including home training. The only difference concerned the assigned respiration rates. In the HRV-biofeedback condition, the resonant frequency was estimated between 4.5 and 6.5 breaths per minute. In the sham condition, respiration rates were chosen from a range that did not target an increase in HRV, i.e., between 10 and 14 breaths per minute. Unfortunately, this is an isolated case, and the use of sham control in HRV-biofeedback research still lacks popularity. 

Moreover, the general majority of studies on HRV-biofeedback do not report the use of any kind of criterion that would evaluate the fidelity of training, i.e., the extent to which it was delivered as intended [50]. Therefore, it is not possible to clearly assess various aspects of training performance, such as understanding and adherence to the instructions, pace and degree of mastering the technique, and, most importantly, the success rate of individual training sessions and the whole intervention. Without universal fidelity measures, it is virtually impossible to compare contradictory results obtained in various studies or make reliable conclusions about the efficiency of training. Similarly, without an explicit criterion, assessment of home training appears problematic. It is commonly assessed based on journal entries, with no possibility to control how the sessions were carried out, or even if at all. An alternative approach relies on built-in assessment algorithms offered by commercial training equipment. Unfortunately, the algorithms used by different companies vary, while the mathematical formulas applied are often not disclosed. All that makes it very difficult to understand and compare the results of training.

A distinct approach to this issue was presented in a study investigating several aspects of the HRV-biofeedback technique and its application in certain disorders and the general population [51,52], where a complex criterion called the Biofeedback Quality Index was proposed. A more straightforward approach was applied in a study comparing the effects of different breathing rates, where participants who did not meet the assigned requirements based on breathing frequency were excluded from the per protocol analysis [53].

### 1.3. Aims of the Study and Hypotheses

Despite a substantial improvement in the controllability of HRV-biofeedback studies published in recent years, most control conditions fail to account for the influence of expectations and involvement. Moreover, when studying the effects of HRV-biofeedback training, it appears crucial to assess the quality of the intervention properly. Since behavioral techniques are acquired by practice, it is even more critical to evaluate the intervention’s fidelity, understood as the methodological strategies used to monitor and enhance the reliability and validity of behavioral interventions [54]. A well-designed fidelity criterion would allow selecting only those participants who truly achieved the coherent/resonant state. In this study, we would like to propose a novel approach aimed at solving these issues. We present a sham paradigm for HRV-biofeedback training intended to mimic the actual intervention accurately.

Moreover, we introduce a simple physiological index for training quality assessment and propose its use as a fidelity criterion for more reliable results. We hypothesize that (1) subjective reception of the sham HRV-biofeedback training by the participants does not differ significantly from the real training. However, we expect that (2) the sham condition does not cause significant changes in resting-state standard HRV parameters as opposed to the real training. Furthermore, we suppose that (3) training quality assessment and application of the fidelity criterion enhance the results’ clarity by increasing the effect sizes of the analyses comparing the effects of real and sham (control) HRV-biofeedback training. To investigate the dose effects of training, we decided to examine between-group differences in training-related changes in resting-state HRV standard measures at two time-points (after 10 and 20 sessions) in comparison to the pre-training baseline.

## 2. Method

### 2.1. Participants

Participants were recruited among students and alumni of the Nicolaus Copernicus University in Toruń as well as from the general working population of young adults. A total of 65 participants enrolled in the study. 60 individuals completed the full training; 4 dropped out after psychometric testing and one after 5 training sessions. Due to signal artifacts, 3 additional participants were excluded. The remaining 57 trainees (30 females, i.e., 52.6%) aged 18–35 (mean = 22.38 ± 3.27) were assigned to one of the two training conditions in a semi-random fashion intended to assure comparable distribution of sex, age, education level, and study major in both groups; 28 participants (14 females, i.e., 50%) aged 19–32 (mean = 22.75 ± 2.98) underwent the real HRV-biofeedback training; 29 volunteers (16 females, i.e., 55.2%) aged 18–35 (mean = 21.93 ± 3.56) were subjected to the sham control treatment. The groups were comparable with respect to the distribution of sex (χ(1) = 0.15, *p* = 0.696, Phi = −0.05), age (U = 312.5, *p* = 0.132), profession/study major, and education level (χ(2) = 4.26, *p* = 0.119, Phi = 0.27). 

Final analysis was carried out on the per protocol sample of 49 trainees, 26 in the real HRV-biofeedback (14 females, i.e., 53.9%) age 19–32 (mean = 22.58 ± 3.02), and 23 in the sham group (12 females, i.e., 52.2%) age 18–35 (mean = 21.70 ± 3.76). Group distributions of sex (χ(1) = 0.01, *p* = 0.907, Phi = 0.02), age (U = 218.0, *p* = 0.101), and education level (χ(2) = 2.585, *p* = 0.275, Phi = 0.225) were comparable. 

Following the methodological guidelines for conducting HRV-related research [26], several confounding variables influencing HRV were considered. Subsequent exclusion criteria were verified by a declaration: practicing professional/extreme sports or dance activities; practicing meditation techniques, yoga breathing exercises, biofeedback training; addictions; psychiatric, emotional, neurodevelopmental, cardiovascular, pulmonary, and/or hormonal disorders; pregnancy. Additionally, habitual levels of alcohol and coffee/tea consumption were controlled for. The participants were instructed to follow a normal sleep routine and refrain from alcohol the day before the experiment, refrain from caffeinated or drinks and meals for 2 h before the experiment, and use the bathroom before the experiment if necessary, and rest in case of arriving in a rush. The study was approved by the Bioethics Committee of the Nicolaus Copernicus University in Toruń at Collegium Medicum in Bydgoszcz. Upon enrollment, each participant provided written informed consent to take part in the study. The document mentioned that training would be carried out in groups differing with respect to the level of feedback-based control and type of visualization used. A monetary reward of 120 PLN (an equivalent of €30) was provided after completing all stages of the study; however, the participants could resign from the study at any given point without giving a specific reason. To compensate for being subjected to a sham condition, the control group would be offered real HRV-biofeedback training. 

### 2.2. HRV-Biofeedback Training Protocol

The HRV-biofeedback training protocol was based on previous work with respect to the number and length of sessions (10 × 20 min) and general instructions adapted for the purpose of this study (abridged and translated from [55]), as well as an introduction of positive emotions [56]. Positive feelings, such as love, happiness, appreciation, or gratitude, were found to improve communication between various systems in the body, thereby increasing HRV [57,58,59,60]. The resonant frequency was not determined beforehand, as the trainees were expected to spontaneously find out their individual pace of breathing during the training. Sessions were scheduled on average, every 1 or 2 days. During the training, the participants were seated in a semi-reclining position. The sessions took place in a well-aerated, air-conditioned room with dimmed lights. The emWave Pro (HeartMath Institute^®^, Boulder Creek, CA, USA) plethysmographic ear sensor and training software were used for the biofeedback training.

The first training session began with a short theoretical introduction to the technique and basic information on the physiological background of HRV-biofeedback. Subsequently, the emWave plethysmographic ear sensor and training software were introduced, while the volunteers were presented their real-time PPG signal in the form of heart rate in beats per minute plotted against time in seconds. Next, the trainees were instructed to pay attention to the following five rules of training: (1) nasal inhalation, prolonged pursed-lips exhalation, (2) diaphragmatic breathing (with instructions to check the way of breathing), (3) natural depth of breathing (to avoid hyperventilation and light-headedness), (4) focus on positive emotions (with instructions to evoke them), (5) improvement of cardiorespiratory coherence (with an introduction to the concept). The state of coherence is a manifestation of enhanced synchronization between breathing and heartbeat. Since it is increased by HRV-biofeedback training, coherence was used in the emWave software as an index of training fidelity, reflecting the level of wave-like regularity of the HRV signal [5]. The coherence value was calculated as peak power (integrated power in a ±0.015 Hz window around the tallest peak within the 0.04–0.24 Hz band) divided by the difference between total power (integrated power within the 0–0.4 Hz band) and peak power, averaged across 64-sec intervals every 5 s. It was presented at the bottom of the screen and visualized in real-time by three color-coded bars in the bottom right corner—green for high, blue for medium, and red for low coherence. The software offered four ‘challenge’ levels (‘low’, ‘medium’, ‘high’ and ‘highest’) of increasing difficulty, characterized by increased low/medium and medium/high coherence thresholds. With increasing difficulty level, higher coherence is necessary to move from the red bar to the blue one and eventually to the green one. The participants were instructed to try to maintain high coherence for as long as possible. Finally, the trainees were comforted not to worry too much about their progress since many factors and aspects of everyday life may influence HRV while worrying and frustration further decrease HRV and interfere with the training [57,58]. The participants were allowed to try out the newly learned technique for a short while before the first session began. 

#### 2.2.1. Real HRV-Biofeedback

For the first few sessions of the real HRV-biofeedback training, the volunteers were offered a pacer (an optional feature available in the software) set to 6 breaths per minute. Since the pacer measured equal time for inhalation and exhalation, the trainees were not meant to follow it closely but rather treat it as a rough estimate of the destined resonant breathing pace, which is far slower than the usual breathing frequency of most people. The pacer was removed later in the course of training when the trainees reported feeling comfortable breathing in their own rhythm. The training progress was based on the coherence values provided by the EmWave software. If progress was substantial (above 90% of high coherence in two subsequent sessions), the difficulty level was raised to make room for further improvements. Some participants never reached ‘high’ difficulty, and no trainee managed to transition to the ‘highest’ level.

#### 2.2.2. Sham HRV-Biofeedback

The sham HRV-biofeedback method was designed to closely resemble real HRV-biofeedback training, yet without the actual feedback signal. All participants received the same introduction and instructions, regardless of the assigned group. However, just before the beginning of the first session, sham trainees were informed that the training would be performed using animations available in the emWave software. These visualizations changed color depending on the current coherence level but were much less intuitive than the HRV plot and did not facilitate following the accelerations and decelerations in heart rhythm. The PPG signal was collected during the sham training, but it was not displayed back to the trainees. Instead, they were presented with animated sessions pre-recorded by the researchers. These sessions were prepared at different levels of difficulty (‘low’, ‘medium’, and ‘high’) and varied with respect to training performance (based on coherence value), which further separated them into subsets of ‘poor’ and ‘good’ training sessions. Sham sessions were presented to the participants in a semi-random order, organized to resemble the course of real training. Each trainee started with sessions pre-recorded at the ‘low’ difficulty level, initially chosen randomly from a subset characterized by low coherence values (‘poor’ performance), and after a few sessions drawn from a subset with better results (‘good’ performance). The difficulty level was raised by the researcher’s decision only after a ‘good’ sham training session (prevalent high coherence in the presented recording). Sham training at the ‘medium’ difficulty and transition to the ‘high’ level occurred in the same manner. However, some sham participants remained at the ‘medium’ level until the end of their training, which was meant to parallel those real HRV-biofeedback trainees who experienced more difficulties mastering the training technique (Figure S1 in the Supplementary Materials). 

### 2.3. Training Expectancy Questionnaire 

In order to evaluate the credibility of the sham intervention, the Training Expectancy Questionnaire (TEQ) was introduced. This self-report tool was constructed for the purpose of this study, partially based on the Credibility/Expectancy Questionnaire (CEQ) [61]. CEQ was devised to assess placebo treatment by examining the trainees’ belief in treatment, measuring the attitude toward rationale (i.e., credibility) and potential benefits (i.e., expectancy). Since all participants were healthy and did not require special treatment, it was necessary to modify the original CEQ. TEQ consists of 10 questions; six answered on a Likert-type scale, three open and one closed-end. The inquiry concerned subjective effects of training on the participants’ health, physical, psychological, and emotional functioning, their expectations and satisfaction with the training, ideas about the aim of the study, and any possible added value of the additional 10 sessions (following the second block of training). TEQ was administered after the 10th and 20th sessions to monitor potential between-group differences in the subjective perception of training. Answers to each question were analyzed separately. Descriptive answers were categorized into nominal variables. Likert-type answers were treated as ordinal variables. The complete questionnaire can be found in Table S1 in the Supplementary Materials. 

### 2.4. Study Design and Timeline

**Figure 1:**
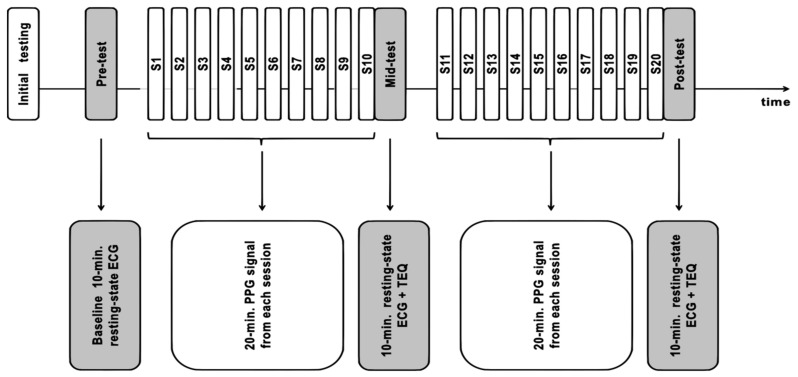
Schematic representation of the study timeline (top row) and measurements taken at the subsequent steps (bottom row). Abbreviations S1 to S20 stand for heart rate variability (HRV)-biofeedback training sessions nos. 1 to 20. Pulse plethysmography (PPG) signal was recorded during HRV-biofeedback sessions. Gray boxes represent psychophysiological data collected at pre-test (baseline), mid-test, and post-test, including resting-state electrocardiography (ECG) and subjective assessment of the training (Training Expectancy Questionnaire, TEQ).

The study was carried out at the Neurocognitive Lab located at the Centre for Modern Interdisciplinary Technologies, Nicolaus Copernicus University in Toruń, Poland, in an artificially-lit, air-conditioned room. The study consisted of 6 major steps: (1) initial questionnaires, (2) pre-test (baseline) psychophysiological measurements, (3) first block of 10 HRV-biofeedback training sessions, (4) mid-test psychophysiological measurements, (5) second block of 10 HRV-biofeedback training sessions, (6) post-test psychophysiological measurements. The timeline of the study is presented as a schematic representation in Figure 1. 

Psychophysiological measurements consisted of psychometric and physiological data collection. Psychometric tests measuring mood, anxiety, and perceived stress levels were administered. Electroencephalography (EEG), ECG, and PPG biosignals were recorded upon 10-min resting-state and three cognitive tasks. The cognitive, psychometric, and neuroimaging aspects will be discussed in a separate report. Blocks of biofeedback training consisted of 10 sessions, each lasting precisely 20 min (for training methods see below), carried out in about two weeks. The last session in each block, i.e., the 10th (S10) and 20th session (S20), was performed with the EEG/ECG sensors and immediately followed by subjective assessment of training with the TEQ questionnaire (see above) and psychophysiological measurements (mid- and post-test, respectively). 

### 2.5. Quantitative Training Quality Assessment with Yield Efficiency of Training Index (YETI)

Development of the training quality index was rooted in the resonant breathing theory, assuming the resonant frequency (RF) breathing rate ranges between 4 and 7 breaths per minute for most humans [55]. This translates to a general RF band in the heart rate frequency power spectrum ranging from 0.067 to 0.117 Hz. The maximum peak of the spectrum should fall into this interval. Most of the power is expected to accumulate in a 0.03 Hz-wide window around the peak frequency, expanding the general RF band by ±0.015 Hz into an interval between 0.052 and 0.132 Hz. The Yield Efficiency of Training Index (YETI) was calculated as stated in Equation (1). It is expressed for each training session as the percent ratio of power in the expanded general RF band (resonance frequency power; RFP) to total power.

(1) $$\mathrm{YETI}\left(i\right)=\frac{\mathrm{RFP}\left(i\right)}{\mathrm{Total}\;\mathrm{Power}\left(i\right)}\times 100\%\;$$

The overall average measure of training quality (YETI_AV_) was calculated by averaging the YETI values across all training sessions (i), where N is the total number of sessions as stated in Equation (2).

(2) $${\mathrm{YETI}}_{\mathrm{AV}}=\frac{1}{N}\overset{N}{\underset{i=1}{\sum }}\mathrm{YETI}\left(i\right)=\frac{1}{N}\overset{N}{\underset{i=1}{\sum }}\frac{\mathrm{RFP}\left(i\right)}{\mathrm{Total}\;\mathrm{Power}\left(i\right)}\times 100\%$$

Additionally, block-average YETI values were calculated separately for each 10-session block as YETI_10_ (i = 1, 2, …, 10) and YETI_20_ (i = 11, 12, ..., 20), allowing for detection of changes in training quality between the two blocks.

The standard deviation of YETI (YETI_SD_) was calculated as the square root of variance in RFP, as stated in Equation (3), where RFP_m_ is the mean value of RFP across all training sessions (i) and N is the total number of sessions.

(3) $${\mathrm{YETI}}_{\mathrm{SD}}=\;\mathrm{sqrt}\;(\frac{1}{N}\overset{N}{\underset{i=1}{\sum }}{\left(\mathrm{YETI}\left(i\right)-{\mathrm{YETI}}_{\mathrm{AV}}\right)}^{2})\;$$

Differences in block averages (ΔYETI) were calculated as simple subtraction (Equation (4)).

(4) $$\Delta \mathrm{YETI}={\;\mathrm{YETI}}_{20}-{\;\mathrm{YETI}}_{10}\;$$

Maximum peak frequency (f_Pmax_) was estimated as the frequency corresponding to the extremum in the power spectrum and averaged across all 20 sessions.

### 2.6. Physiological Data Analysis

**Figure 2:**
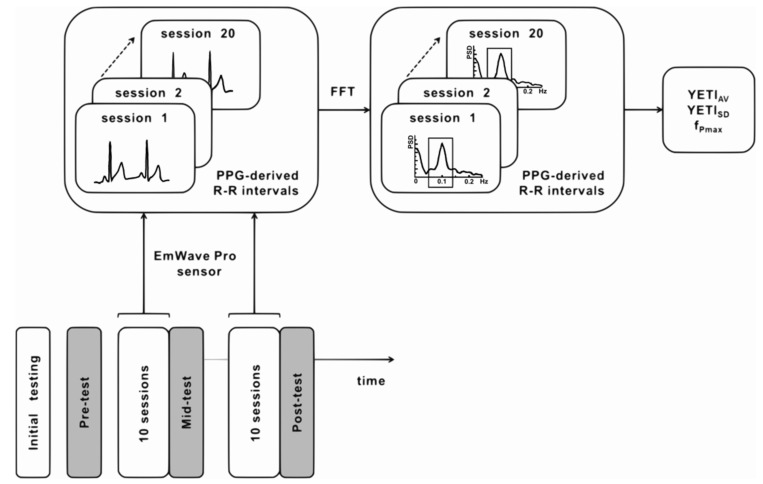
Schematic representation of the analytic pipeline applied to PPG data collected from each participant during HRV-biofeedback training session.

Data collected during HRV-biofeedback training sessions (Figure 2) and resting-state psychophysiological measurements (Figure 4) were analyzed separately with different methods and for distinct purposes.

#### 2.6.1. HRV-Biofeedback Training Data 

During the training sessions, the plethysmographic signal was recorded from the left ear lobe using the emWave Pro ear sensor (HeartMath Institute^®^, Boulder Creek, CA, USA) at a 370 Hz sampling rate. A schematic representation of PPG data processing is presented in Figure 2. RR series (beat-to-beat intervals) was exported from emWave software and analyzed in Spyder (Python). The RR intervals were interpolated using the cubic spline method at the rate of 4 Hz. The FFT-based power spectrum was calculated using Welch’s periodogram method with a window width of 256 s and 50% window overlap. For each session of each participant, a spectral profile and values of YETI and f_Pmax_ were calculated. The overall training performance of each participant was assessed by three parameters calculated across all 20 sessions: YETI_AV_, YETI_SD,_ and average f_Pmax_. 

#### 2.6.2. YETI-Based Clustering and the Fidelity Criterion 

**Figure 3:**
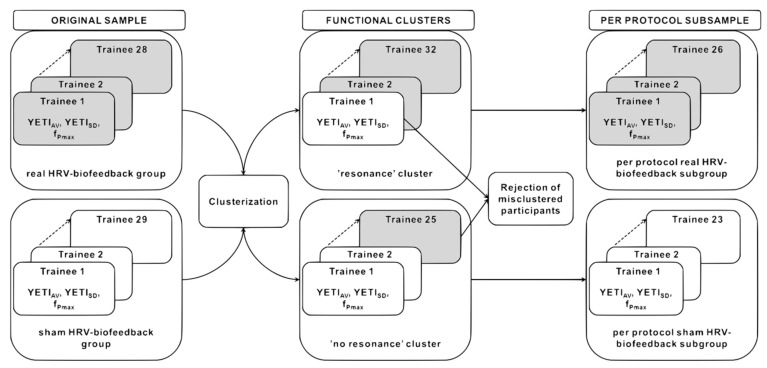
Schematic representation of participant clustering and exclusion procedures based on training performance parameters (YETI_AV_ and YETI_SD_). The participants are separated into two clusters based on similarity in the physiology of their training performance, irrespectively of their originally assigned experimental condition (real vs. sham HRV-biofeedback group, 28 and 29 trainees, respectively). Clustering results in two functional clusters, characterized by participants exhibiting ‘resonance’ and ‘no resonance’ upon training (32 and 25 trainees, respectively). In the last step, misclustered participants are rejected, and only participants training per protocol remain in the per protocol real and sham HRV-biofeedback subsamples (26 and 23 trainees, respectively).

Next, training fidelity was assessed in order to identify those participants who trained per protocol. This per protocol subsample was selected based on their training performance parameters, YETI_AV_ and YETI_SD,_ using two-step cluster analysis (Figure 3). This method is a hybrid approach combining a distance measure that separates groups (pre-clustering), followed by a probabilistic approach that chooses the optimal subgroup model (clustering). In the first step, data are pre-clustered in a sequential approach based on dense regions defined in the analyzed attribute space. In the second step, pre-clusters are statistically merged in a stepwise fashion up to the point when all belong to one cluster. The main advantages of this method include determining the number of clusters based on a statistical measure of fit (Akaike information criterion (AIC) or Bayes information criterion (BIC)) instead of an arbitrary choice, using categorical and continuous variables simultaneously, analyzing outliers, and handling large datasets. It is also one of the most reliable in terms of the number of clusters detected, classification probability, and reproducibility [62]. Two-step clustering was performed in SPSS statistical software version 25.0 (IBM Corp, Armonk, NY, USA, 2012). Based on the similarity of training performance parameters, participants were separated into two functional clusters: (1) those exhibiting and (2) those not exhibiting cardiovascular resonance during biofeedback training sessions (Figure 3). Real biofeedback trainees were expected to fall into the ‘resonance’ cluster and sham participants into the ‘no resonance’ group. The misclustered individuals were rejected as not training per protocol. The fidelity criterion demanded evoking a resonant state in the real biofeedback and not evoking it in the sham condition.

#### 2.6.3. Resting-State HRV Data 

**Figure 4:**
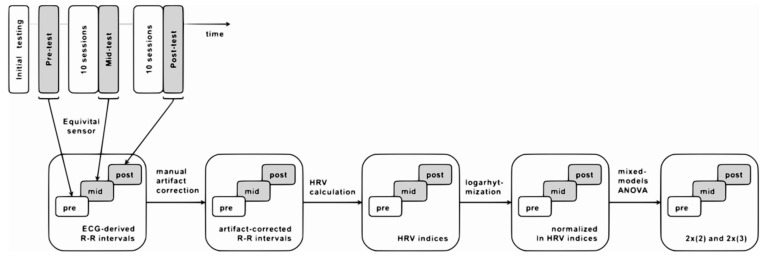
Schematic representation of the analytic pipeline applied to ECG data collected from each participant during resting-state psychophysiological measurements.

Resting-state electrocardiographic activity was recorded at a 256 Hz sampling rate during pre-, mid-, and post-test using telemetric belts (Equivital Inc., New York, NY, USA). A schematic representation of ECG resting-state data processing is presented in Figure 4. The RR intervals were derived via internal software of the device and exported for analysis in Kubios HRV v2.0 software (University of Eastern Finland). Artifacts were corrected manually, and standard HRV indices were calculated using both time- and frequency-domain methods. The following HRV measures were chosen to evaluate the effect of training: standard deviation of the NN, i.e., normal RR intervals (SDNN), root mean square of the standard deviation of the NN (RMSSD), the total power of the signal (TP; 0–0.4 Hz), as well as power in standard frequency bands: low-frequency (LF; 0.04–0.15 Hz) and high-frequency (HF; 0.15–0.4 Hz). These indices were selected based on two criteria: (1) popularity and recommendations in the HRV-biofeedback literature [23] and (2) coverage of different aspects of human physiology [26]. Choosing the most commonly used parameters allows for broad cross-study comparisons. At the same time, each of the selected parameters has a different physiological meaning. The SDNN reflects the cyclic components responsible for HRV. The LF power provides information on both sympathetic and vagal activity, as well as on the functioning of the baroreflex. RMSSD and HF power both reflect the vagal tone; however, RMSSD is relatively free of respiratory influences and therefore complements the results of the frequency analysis [26]. Total power encompasses the complete variance of the signal across all the frequencies and provides a general picture of the magnitude of the HRV fluctuations [23]. Prior to spectrum estimation, cubic spline interpolation of RR series was performed at the rate of 4 Hz with 256 points in the frequency domain. FFT was calculated using Welch’s periodogram method with a window width of 256 s and 50% window overlap. To assure normal distributions, the HRV indices were subjected to logarithmic transformation. 

### 2.7. Statistical Data Analysis

Statistical analysis was performed in SPSS statistical software version 25.0 (IBM Corp, 2012), statistical significance was assumed at *p* < 0.05. The effect size was calculated as partial eta-square (η_p_^2^). The credibility of Sham HRV-biofeedback was analyzed by comparing TEQ answers between real and sham HRV-biofeedback groups using the Chi-square test. For each training condition (real and sham training), between-block changes in YETI values (ΔYETI) were calculated using the Wilcoxon rank test. Between-group differences in training quality were calculated by comparing YETI_AV_, YETI_10,_ YETI_20,_ and ΔYETI values with the Mann–Whitney test. Baseline values of the HRV indices (from pre-test resting-state recordings) were compared between the real and sham HRV-biofeedback groups using the Mann–Whitney test.

Effects of the type of HRV-biofeedback training (real training vs. sham control) on resting-state HRV were calculated with mixed-models analysis of variance (ANOVA), separately for each HRV measure (lnSDNN, lnRMSSD, lnTP, lnLF, lnHF). Two models were calculated for each parameter. The first model included the two extreme time-points (pre-post) in order to investigate the effect of the whole training period (total effect of 20 sessions). The second model used all three time-points (pre-mid-post) to analyze the dose-effect. 

The effects of the type of training as described above were investigated on three data sets: the original sample, the functional clusters, and the per protocol subsample. Comparing the results obtained from the initial sample with those from the functional clusters was intended to support the notion that based on their physiology, the misclustered subjects should not belong to the group they were assigned to, but rather the other condition. Results obtained from the initial sample and from the per protocol subsample were compared in order to show the benefits of applying the fidelity criterion. To address differences in sample sizes, bias-corrected and accelerated (BCa) bootstrap intervals were calculated for all simple effects tests using random sampling bootstrapping based on 10,000 samples. Inflation of the alpha error due to the family-wise error rate in the post-hoc analysis was addressed by applying the Bonferroni correction. As advised by Armstrong [63], no correction was applied against the experiment-wise error rate due to the following circumstances: the study was restricted to a small number of planned comparisons, the results of the individual tests were of main interest (instead of a common null hypothesis), and the investigated dependent variables were not independent of each other (as all described cardiovascular functioning). Moreover, the focus of this study was much more heavily placed on differences in results between data samples rather than on the results themselves. At the same time, due to limitations presented by conclusions drawn based on *p*-values [64], increased interest was invested in comparing effect sizes and test power. Nevertheless, for reasons of methodological scrutiny, the Benjamini–Hochberg procedure [65] was applied to the results of the interaction between training and group. The critical value was calculated for a false discovery rate (FDR) of 0.05 and 0.1; significant interactions denoted by two asterisks (**) and one asterisk (*), respectively (Table 2). 

## 3. Results

### 3.1. Clustering of Training Data and the Fidelity Criterion

**Figure 5:**
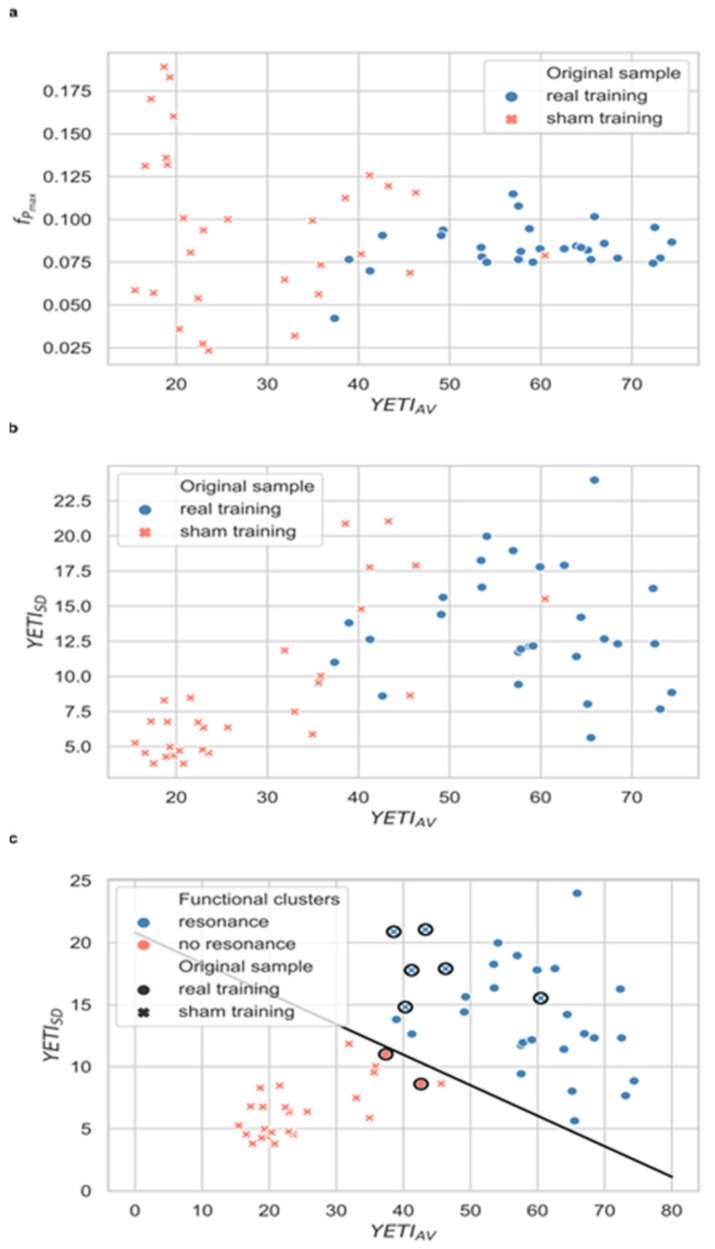
Scatterplots of YETI_AV_ values against f_Pmax_ (**a**) and YETI_SD_ (**b**,**c**). Plots (**a**,**b**) show the original data set (original sample). Markers represent the training conditions: real (blue dots) and sham (salmon exes) HRV-biofeedback. Plot (**c**) depicts the results of dividing the data set into functional clusters. The original sample training data, i.e., real biofeedback (dots) and sham (exes) were divided into 2 clusters: resonance (blue) and no-resonance (salmon). Misclustered participants (salmon dots and blue exes; circles around markers) were excluded from the per protocol subsample. The border between the two clusters is defined by the following equation: y = −0.25x + 20.81.

The differences in training quality between the two experimental groups were depicted by plotting the individual values of YETI_AV_ against the maximum peak frequency (f_Pmax_) averaged across the 20 sessions. The real HRV-biofeedback group exhibits higher YETI_AV_ and f_Pmax_ located within the RF band, while sham trainees are characterized by lower YETI_AV_ values and f_Pmax_ often out of the RF band (Figure 5a). Another useful auxiliary measure is the standard deviation of YETI (YETI_SD_), showing changes in YETI values over time, similar in interpretation to a learning parameter. Plotting the individual values of YETI_AV_ against YETI_SD_ shows that the real HRV-biofeedback trainees improved during the course of training. At the same time, the sham biofeedback group exhibited stable (and low) values of YETI across all 20 sessions (Figure 5b). 

In order to separate successful and failed training (and control) a two-step clustering based on YETI_AV_ and YETI_SD_ values was performed. The majority of real HRV-biofeedback trainees were classified into the ‘resonance’ cluster (26 out of 28, 92.86%), while most volunteers subjected to sham training—into the ‘no resonance’ group (23 out of 29, 79.31%). A total of 8 participants were misclustered (i.e., assigned to a different cluster than the rest of their original group); 2 from the real HRV-biofeedback group, and 6 sham trainees (Figure 5c). Their data were excluded from the per protocol subsample.

### 3.2. Credibility of Sham HRV-Biofeedback

After the first 10 sessions, the real HRV-biofeedback trainees reported an influence on their physical functioning significantly more frequently than the sham control (N_real_ = 9 (32.1%), N_sham_ = 3 (1.3%); χ(1) = 4.073 *p* = 0.044, φ = −0.267), while the opposite was observed for feeling tired during the training (N_real_ = 1 (3.6%), N_sham_ = 7 (24.1%); χ(1) = 4.994, *p* = 0.025, φ = 0.296). After completing the whole training (20 sessions) the only significant difference concerned significantly more frequent reports of an influence on mental functioning in the HRV-biofeedback group (N_real_ = 20 (74.1%), N_sham_ = 13 (44.8%); χ(1) = 4.941, *p* = 0.026, φ = −0.297). 

In the per protocol subsample, after the first 10 sessions no significant differences were noted. The difference in perceived influence on mental functioning after 20 sessions remained statistically significant (N_real_ = 20 (8.0%), N_sham_ = 9 (39.1%); (χ(1) = 8.369, *p* = 0.004, φ = −0.418). Moreover, participants involved in the sham training significantly less frequently admitted any additional benefit of the extra 10 sessions (N_real_ = 15 (88.2%), N_sham_ = 10 (5.0%); χ(1) = 6.130, *p* = 0.013, φ = −0.407).

### 3.3. Quantitative Measures of Training 

For each training condition (real and sham training), between-block changes in YETI values (ΔYETI) were non-significant, both in the original sample (Z_real_ = −0.71, *p* = 0.480; Z_sham_ = −0.44, *p* = 0.658), and in the per protocol subsample (Z_real_ = −0.52, *p* = 0.603; Z_sham_ = −0.15, *p* = 0.879). Between-group comparison of YETI values averaged across blocks (YETI_10_ and YETI_20_) and the whole training period (YETI_AV_) revealed significant differences, while values of ΔYETI did not differ between groups. Similar results were obtained for the original sample and the per protocol subsample (Table 1).

**Figure 6:**
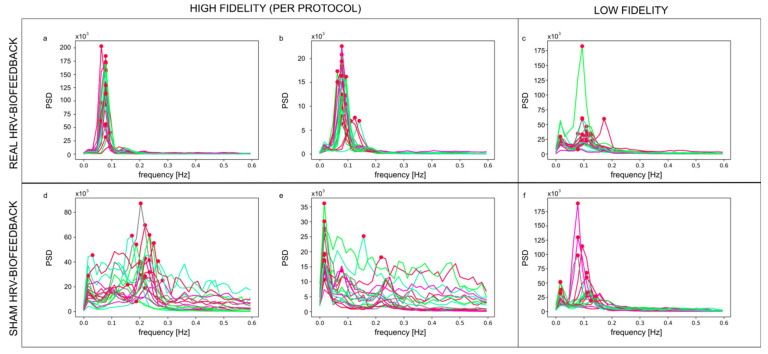
Sets of 20 spectral profiles (one line for each session) for 6 participants initially assigned to the real (**a**–**c**) and sham (**d**–**f**) HRV-biofeedback training. The dots mark the maximum peak for each profile. Notice the difference in power amplitude (scale of power spectrum density; PSD) and concentration/dispersion of individual peak frequencies between graphs, which differentiate training per protocol (high fidelity (**a**,**b**)) from low fidelity training (**c**), and proper control (**d**,**e**) from failed (low fidelity) sham condition (**f**).

Spectral profiles of the PPG-derived RR signal collected upon training differed between the real and sham HRV-biofeedback conditions. For most of the real biofeedback trainees, the power spectrum density (PSD) of the signal was concentrated in a narrow band around the maximum peak. The peak frequency was consistent across the 20 sessions (Figure 6a,b). The majority of the sham control group exhibited a lack of PSD concentration and peak frequency changing from session to session (Figure 6d,e). A few participants showed profiles atypical for the condition they had been initially assigned to. Displaying a pattern characteristic for the opposite condition indicated instances of failure in adherence to the training or control conditions (Figure 6c,f, respectively), i.e., low fidelity of training. 

### 3.4. Effects of Training

No significant baseline differences between real and sham HRV-biofeedback for resting-state SDNN, RMSSD, TP, LF, and HF were found, either in the original sample (*p*-values equal to 0.330, 0.231, 0.434, 0.962, and 0.160, respectively) or in the per protocol subsample (*p*-values equal to 0.100, 0.060, 0.144, 0.389, and 0.078, respectively; for full results see Table S2 in the Supplementary Materials).

The effects of training were analyzed with two models. The total effect of the full training period was calculated on data collected before and after all 20 sessions (pre-post). The dose-effect of training was investigated by comparing data from 3 time-points (pre-, mid-, and post-test). Both models were tested on (a) the original sample, (b) the functional clusters, and (c) the per protocol subsample. The interaction effect between group and training for each of the aforementioned conditions is presented in Table 2. 

#### 3.4.1. Total Effect

Results of the pre-post analysis showed no significant between-subject differences (no main effect of the group) for all five HRV indices in all samples.

Within-subject analysis on the original sample revealed a significant main effect of training for lnSDNN, lnRMSSD, lnTP, lnLF, and approaching significance for lnHF. The interaction effect was significant for lnSDNN (F(1,55) = 4.50, *p* = 0.038, η_p_^2^ = 0.08, pow = 0.55), lnTP (F(1,55) = 5.64, *p* = 0.021, η_p_^2^ = 0.09, pow = 0.65), and lnHF (F(1,55) = 4.75, *p* = 0.034, η_p_^2^ = 0.08, pow = 0.57). Post-hoc analysis of the interaction effects for lnRMSSD, lnTP, lnLF, and lnHF revealed that the effect of training was statistically significant only in the real biofeedback group (*p*-values equal to 0.003, <0.001, 0.020, and 0.010, respectively), as opposed to the sham control (*p*-values equal to 0.359, 0.093, 0.421, and 0.814, respectively). Only for lnSDNN the results were significant in both groups (*p*-value equal 0.002 for real, and 0.027 for sham HRV training).

Within-subject analysis repeated on functional clusters yielded similar main effects of training but stronger interaction effects. For all considered HRV indices, higher η_p_^2^, lower *p*-value, increased F-value and power were observed, and all interactions reached statistical significance. 

Within-subject analysis repeated on the per protocol subsample showed a significant main effect of training for lnSDNN, lnRMSSD and lnTP, and approaching significance for lnLF. Interaction effects were present for all indices, again stronger than for the original sample with higher η_p_^2^, lower *p*-value, increased F-value, and power (lnSDNN: F(1,47) = 6.56, *p* = 0.014, η_p_^2^ = 0.12, pow = 0.71; lnRMSSD: F(1,47) = 5.67, *p* = 0.021, η_p_^2^ = 0.11, pow = 0.65; lnTP: F(1,47) = 7.75, *p* = 0.008, η_p_^2^ = 0.14, pow = 0.78; lnLF: F(1,47) = 5.74, *p* = 0.021, η_p_^2^ = 0.11, pow = 0.65; lnHF: F(1,47) = 6.07, *p* = 0.017, η_p_^2^ = 0.11, pow = 0.68). In the per protocol subsample differences in group means increased for all parameters (lnSDNN, lnRMSSD, lnTP, lnLF, and lnHF), and significant effects of training were observed only in the real biofeedback condition (*p*-values equal to 0.002, 0.003, 0.001, 0.021, and 0.010, respectively), in contrast to the sham control (*p*-values equal to 0.415, 0.932, 0.332, 0.597, and 0.535, respectively). 

Detailed results of ANOVA and post-hoc analysis are presented in the Supplementary Materials in Tables S3 and S4, respectively.

#### 3.4.2. Dose Effect

Data analysis at 3 time-points (pre-mid-post) revealed no significant main effect of group for any of the analyzed HRV measures in any data set. 

The main effect of training was significant or approaching significance across all data sets for all HRV indices except lnHF. 

The interaction effect in the original sample was approaching significance for lnTP (F(2,110) = 2.76; *p* = 0.07; η_p_^2^ = 0.05; pow = 0.535) and lnHF (F(2,110) = 2.81; *p* = 0.065; η_p_^2^ = 0.05; pow= 0.54) and insignificant for lnSDNN, lnRMSSD and lnLF. Post-hoc analysis showed significant increases between pre-test and mid-test for lnSDNN and lnTP both in the real biofeedback (*p* = 0.021 and 0.019, respectively) and sham group (*p* = 0.006 and 0.023, respectively). 

The interaction effect in the functional clusters showed significant interactions for lnSDNN, lnTP, lnLF, while for lnHF the relation remained approaching significance. 

The interaction effect in the per protocol subsample was significant for lnTP (F(2,94) = 3.68; *p* = 0.029; η_p_^2^ = 0.07; pow = 0.66), lnLF (F(2,94) = 3.48; *p* = 0.035; η_p_^2^ = 0.07; pow = 0.64) and lnHF (F(2,94) = 3.14; *p* = 0.048; η_p_^2^ = 0.06; pow = 0.59), and approaching significance for lnSDNN (F(2,94) = 2.79; *p* = 0.066; η_p_^2^ = 0.06; pow = 0.54) and lnRMSSD (F(2,94) = 2.70; *p* = 0.072; η_p_^2^ = 0.05; pow = 0.52). Post-hoc analysis revealed an increase in lnSDNN and lnTP values from pre-test to mid-test in the real biofeedback (*p* = 0.028 and 0.030, respectively) and sham group (*p* = 0.019 and 0.043, respectively). Changes for lnLF and lnHP approached significance. Values of lnLF increased between pre- and mid-test in the real training group (*p* = 0.067), while both lnLF and lnHF decreased between mid- and post-test in the sham control (*p* = 0.060 and 0.065, respectively). 

**Figure 7:**
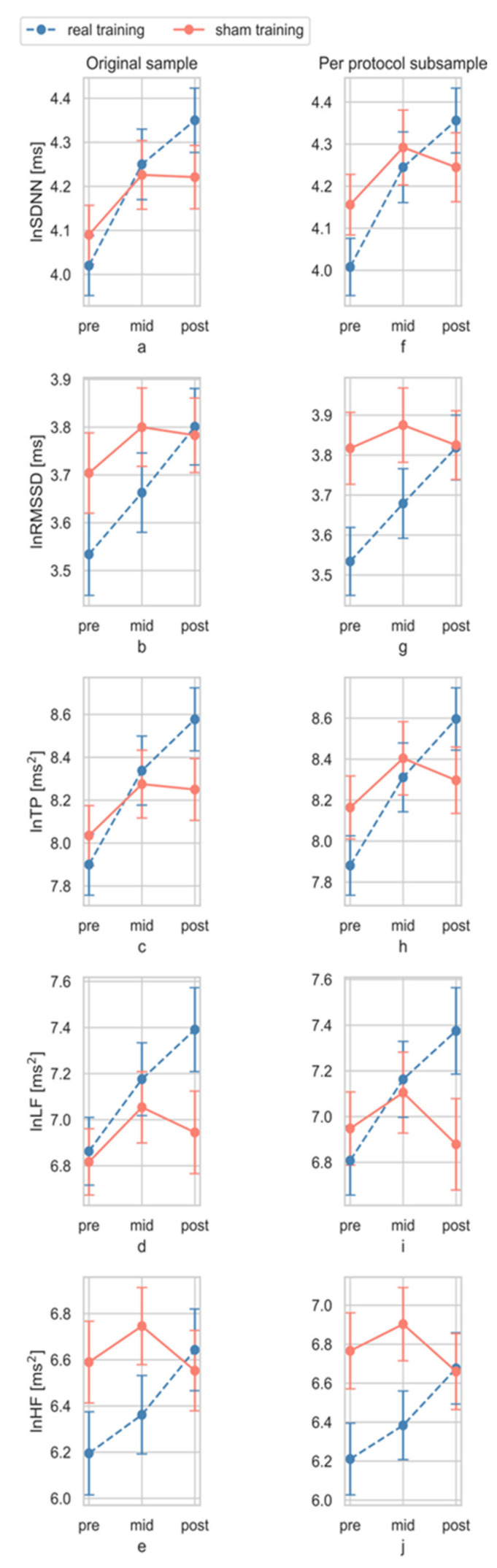
Effects of real (blue) and sham (salmon) HRV-biofeedback training on HRV parameters: lnSDNN (**a**,**f**), lnRMSSD (**b**,**g**), lnTP (**c**,**h**) lnLF (**d**,**i**) and lnHF (**e**,**j**). Real HRV-biofeedback (blue) is compared to the novel sham control (salmon) in the original sample (left column) and the per protocol subsample (right column) at 3 time-points: before the intervention (pre-test), after 1 block of training, i.e., 10 sessions (mid-test), and after 2 blocks, i.e., 20 sessions (post-test). Error bars show standard error of the mean (SEM).

Graphs illustrating these findings are presented in Figure 7. Detailed results of ANOVA and post-hoc analysis are shown in the Supplementary Materials in Tables S5 and S6, respectively.

## 4. Discussion

In this study, two practical and, in our opinion, vital problems were tackled by applying novel tools. The sham HRV-biofeedback training is a good control condition, subjectively indistinguishable from the real training, yet it does not improve resting-state HRV, as the real training does. Assessment of training fidelity based on YETI, an objective index of training quality, allowed for a more specific and sensitive analysis of the collected data with respect to a general effect of training, expressed by increased effect size. Comparison of data collected after 10 and 20 sessions revealed a dose-effect of training. We conclude by discussing the limitations of our study approach. 

### 4.1. Novel Sham HRV-Biofeedback Training

In this study, a novel sham training protocol was introduced as a control condition for the HRV-biofeedback method. To the best of our knowledge, no sham HRV-biofeedback training involving fake signal being fed back to the participants has been conducted up to date. The control conditions closest to our design were normal-pace breathing biofeedback [66] and training at respiration rates not affecting HRV (10–14 breaths per minute) [49]. Nevertheless, resonant effects of HRV frequencies harmonic to 0.1 Hz, such as 0.2 Hz, present during natural breathing, have not been specifically investigated. Moreover, paced respiration training appears to influence HRV measures irrespective of breathing frequency [66]. Our sham feedback signaled to the participants the need for an adjustment in their breathing frequency at random moments, preventing regular respiratory patterns from being maintained for prolonged periods of time.

Apart from the feedback signal source, another major difference between real and sham HRV-biofeedback was the type of visual stimuli used to present the biofeedback information. This difference in visual feedback is a minor yet necessary drawback since the two conditions had different training requirements. A linear HRV plot was shown during the real training, as it was easy to understand and follow. Unfortunately, the presentation of a pre-recorded HRV signal in this form to the sham trainees allowed them to synchronize their breathing with the presented feedback, resulting in unwanted training. On the other hand, color-coded simple animations displayed in the sham condition were less straightforward and did not suggest any breathing pattern. However, training with animations was not intuitive enough for the real biofeedback practice, causing problems in mastering the technique (pilot study data, unpublished). Therefore, we decided to apply different animation types in the two groups that would be most suitable for the purpose intended.

Although the two experimental conditions were almost identical in design, the credibility of the sham control was assessed. Subjective reception of both kinds of training was investigated with the Training Expectancy Questionnaire, applied at the mid- and post-test (after 10 and 20 sessions, respectively). Comparing the TEQ results between the two conditions revealed similar experiences of the training itself and analogous perception regarding the subjective effects of training on health, physical, mental, and emotional functioning. Presented levels of awareness about the study and experimental hypotheses were also comparable between conditions. The significant difference found in the per protocol subsample concerned the assessment of the additional 10 sessions, which were perceived as beneficial significantly more often by real HRV-biofeedback trainees. This finding most likely reflects the usefulness of proper cues in the real biofeedback, allowing for improvement and learning, and the inconsistency of the sham signal, causing certain hardship in interpretation and preventing the subject from acquiring an ability to self-regulate breathing at any distinct pace.

### 4.2. Training Quality and Applicability of the YETI Index

Although the two types of training were almost identical in subjective reception, the two groups exhibited significant differences in training quality. The spectral profiles derived from the training sessions showed distinct maximum peaks within the resonance frequency band (0.067–0.117 Hz) during real HRV-biofeedback sessions, while power was much more evenly spread across all physiological frequencies in the sham condition (Figure 6). Quantitative comparison of these differences was possible thanks to the YETI index. Its values averaged across all sessions (YETI_AV_) were significantly different between groups already after the first block of training, and this difference further increased after the second block. Plotting the values of YETI_AV_ against the average maximum peak (f_Pmax_) for each participant visualized individual proportions of power within the RF band vs. the most common frequency present along the training. As expected, real biofeedback training was characterized by higher values of YETI_AV_ and f_Pmax_ within the RF band, while sham training exhibited lower YETI_AV_ values and f_Pmax_ spread across a wide range of frequencies, rarely within the RF band (Figure 5a). However, these two parameters are both based on the concept of the RF band, and as such, carry information that is correlated and redundant for clustering purposes. Across-session standard deviation of YETI values (YETI_SD_), on the other hand, adds information on the change in training quality. Small variations in YETI reveal relatively stable performance, while large YETI_SD_ may be used as an approximation of learning (Figure 5b).

The two conditions (real vs. sham training) were separated accurately with the two-step clustering method based on YETI_AV_ and YETI_SD_ (Figure 5c). Analysis of the spectral profiles shows that the observed cases of misclustering were justified and reasonable. Real HRV-biofeedback trainees assigned to the ‘no resonance’ cluster exhibited low power of the signal and/or shifted peak maxima (Figure 6c), similar to those observed during sham sessions. On the other hand, sham participants clustered as ‘resonance’ condition displayed high power of the signal and clear, stable peaks within the RFB (Figure 6f), typical for real HRV-biofeedback training. The physiology of these misclustered participants did not behave in a predicted manner, revealing a low fidelity of training. It is known that biofeedback techniques are challenging to some people [55]. Nevertheless, when discussing specific physiological effects of an intervention, only results of high-fidelity training should be considered [50]. Cases of resonance training in the sham group most probably arose from indifference to the false feedback occurring in participants who relied more on their internal perception of breathing than on the presented visualization. As reported previously, physiological effects of slow-pace breathing are almost identical, only slightly weaker than those of HRV-biofeedback [41]. 

For these reasons, the misclustered trainees were not included in the per protocol analysis. Clustering results were further supported by between-group analysis of five HRV indices (SDNN, RMSSD, TP, LF, and HF). In comparison to the original groups as assigned, ANOVA results obtained from the functional clusters and per protocol subsample showed increased effect sizes, significance, and test power.

### 4.3. Quantitative Effects of Training

The total effect of the complete training period, analyzed by comparing changes in resting-state HRV from baseline (pre-test) to post-test, differentiated real HRV-biofeedback training from the novel sham control condition. For all five HRV measures used (SDNN, RMSSD, TP, LF, and HF), most of the effect sizes (η_p_^2^) were small or medium. It is likely that the rather small improvements in HRV could be attributed to the ceiling effect. In a homogenous group of educated individuals, it could be difficult to perceive HRV changes [67]. Moreover, for young, healthy adults, there might be little room for improvement in psychophysiological functioning [68]. Another probable cause could be the influence of individual differences in response to [15]. These may stem from age, race, and sex differences, or the impact of baseline HRV, among other factors [69]. The interaction effect was attenuated for l nHF due to baseline differences between groups (main group effect). However, the impact of training on HRV was still noticeable, as the value of lnHF increased at post-test in the real biofeedback group.

With respect to the original groups, the analysis performed on the YETI-based functional clusters and the per protocol subsample showed stronger effects of the type of training. Interaction analysis yielded larger effect sizes (η_p_^2^), F-values, and test power. The *p*-values were lower, often resulting in a situation where a test that was non-significant in the original sample reached significance in the functional clusters and the per protocol subsample (Tables S3, S4 and S6). It is very noteworthy that the highest values were observed for the functional cluster, suggesting that, indeed, the mis-categorized participants were clustered correctly by the algorithm, and their physiology behaved in line with the training condition that they were not assigned to.

The literature suggests that four sessions are sufficient to observe the effects of HRV-biofeedback training [70]. We analyzed the dose-effect of training in a more detailed approach, considering data from three time-points (Figure 7). The interaction effects of group and training were maintained. However, the first 10 sessions resulted in similar effects in both groups. In contrast, the second block of training resulted in an increase of HRV in the real biofeedback group and a slight decrease in the sham group. The initial improvement of HRV measures in the control condition could not be attributed to cardiovascular resonance since the sham group achieved significantly lower block-average YETI values (YETI_10_ and YETI_20_) than the real biofeedback group. Moreover, for both conditions, block-average YETI values did not differ between blocks. Concentrative and meditation-like effects [71,72] and positive emotions [56] could influence the levels of HRV in the initial phase and act similarly to HRV-biofeedback concerning stress reduction [73]. Furthermore, HRV can be influenced by the placebo effect, as it is known that autonomic recovery can be enhanced by a placebo suggestion [74] and cognitive expectancy [75]. Therefore, an influence of the placebo effect caused by sham training is a very likely explanation for improvements of HRV values in the control group at the initial stages of training. Subsequently, a decrease of all HRV indices in the direction of baseline was observed after the second block of training for the sham group. This drop in HRV could reflect a loss of interest, habituation, or frustration with the training [57,58]. A parallel effect was observed in subjective measures of the training reported on the TEQ questionnaire. The initial 10 sessions appear to be experienced by both groups in a similar way, but after the second block of intervention real biofeedback trainees report an influence of HRV-biofeedback on their mental functioning significantly more often, which might point to perceived psychological benefits of the training.

### 4.4. Beyond HRV-Biofeedback

Although this study was conducted in the HRV-biofeedback paradigm, we believe that the topics presented in our manuscript could be of great importance to the whole biofeedback community and beyond. Methodological problems tackled in this study—introducing an appropriate control condition that would address the placebo effect, controlling the effects of training in a quantitative manner, and assessing treatment fidelity—are crucial and central to any behavioral intervention. Sham control is gaining popularity not only in biofeedback research, where it was applied in studies using neurofeedback [43,46] or biofeedback based on electromyography (EMG) [76,77]. Recently, sham treatments were reported for repetitive transcranial magnetic stimulation (rTMS) [78] and mindfulness meditation [79,80,81]. Moreover, a psychophysiological aspect is involved in most behavioral treatments, such as changes to HR and HRV upon mindfulness [81] and heartfulness [82] meditation training. Various types of meditation may evoke different effects on the cardiovascular system [83]. Moreover, different effects of concentration meditation depend on individual maximal frequency peaks achieved by each participant [84]. Therefore, it would be feasible to quantify the results of these types of intervention based on physiological indices, allowing for more effective application and guidance during training. 

YETI or similar training quality indices based on other physiological measures could be very helpful for quantitative quality control upon application of these techniques. Likewise, assessing the fidelity of an intervention is highly recommended, yet often very heterogeneous, as in the case of behaviour change interventions that promote physical activity [85]. Robust systems of fidelity evaluation are needed for complex behaviour change interventions to evaluate intervention integrity [86]. The topic was highly crucial and received the attention of the Treatment Fidelity Workgroup of the National Institutes of Health Behavior Change Consortium. They developed a comprehensive model of treatment fidelity covering five factors of treatment: study design, providers, delivery, receipt, and enactment skills [87]. This model was tested in a case example study of an exercise intervention where fidelity assessment was based on observation and self-report; nevertheless, evidence of treatment fidelity to training was not quantified [54]. The computational methods proposed in our manuscript provide a means to evaluate treatment fidelity based on measurements and hard data. Implementation of this methodology for behavioral interventions other than biofeedback could substantially aid the development and application of these techniques. 

### 4.5. Limitations and Further Studies

Given a large number of analyses and thus increased risk of alpha error, the main limitation of this study was a relatively small sample size. This was partially addressed by performing bootstrapping on the ANOVA tests. Moreover, the sample was very homogeneous and, therefore, the results should be extrapolated to other age and ethnic groups very carefully. 

Although no significant differences in baseline HRV levels were found between the two groups, a main effect of the group overshadowed the influence of the type of training on the HF parameter (non-significant interaction effect). During the training sessions and in the resting-state condition, breathing was not controlled for, excluding the possibility to correct the HRV parameters for respiration. For this reason, the existence of group-wise variations in resting-state respiration patterns and their possible influence on differences in HRV parameters cannot be excluded. Further studies should be carried out on groups with very similar baseline HRV levels and control for respiratory parameters. Moreover, a few confounding factors suggested by Laborde and colleagues [26] were not controlled for, including weight, height, and waist-to-hip ratio, oral contraceptive intake for female participants, as well as intense physical training the day before the experiment, potentially influencing the experimental results. Furthermore, the addition of a no-intervention group could help to confirm the probable influence of the placebo effect at the initial stage of sham training.

The aim of this study was to propose solutions to certain methodological problems present in the field. This report highlights the necessity to control training quality in HRV-biofeedback research, as well as attempts to provide a reliable index of training fidelity. In the present form, the applicability of the presented method is limited due to its dependence on a particular dataset. However, the authors plan to apply machine learning classification algorithms to improve their method further to make it robust and easily applicable. The proposed novel sham HRV-biofeedback appears to be a generally good control condition, although a few trainees presented characteristics of cardiovascular resonance upon sham training. Nevertheless, using other control conditions does not exclude similar behavior—participants may evoke cardiovascular resonance spontaneously and unconsciously during any type of control intervention. This could happen for various reasons, such as induction of deep relaxation or concentration approaching meditative states, self-invoked resonance breathing, etc. Therefore, it appears crucial to apply a training fidelity index not only to real biofeedback training but also to control conditions. 

In order to address the potential influence of individual differences on the effects of training, further analysis will take into consideration additional confounding factors, such as sex, temperamental features, and baseline values of anxiety, mood, and HRV. Moreover, future studies will explore the interactions between HRV and brain activity in an attempt to shed some light on the mechanisms behind the effects of HRV-biofeedback training.

Last but not least, it would be beneficial to apply the principle of quantitative physiology-based training quality index and fidelity assessment presented in this study to different biofeedback modalities and other types of behavioral training, such as mindfulness meditation. 

## 5. Conclusions

The research field of HRV-biofeedback needs clear methodological standards regarding training protocols, equipment, session timing (duration, frequency, number, etc.), standard measures taken, and HRV indices analyzed. The novel sham HRV-biofeedback training condition proposed in this study appears to be a credible control, not distinguishable in reception as a form of training, yet addressing the possible placebo effect. The YETI index is useful to assess the individual quality of training and fidelity of intervention (both real HRV-biofeedback training and sham control). Participant selection according to the fidelity criterion based on the average YETI index and its standard deviation (YETI_SD_) permits analysis to be conducted on more homogeneous data. Application of YETI allows for more informed description and examination of collected data and yields results of higher specificity and reliability, reflected by increased effect size and improved statistical significance. Any behavioral treatment affecting human psychophysiology would most likely benefit from introducing a quantitative, physiology-based index and controlling training quality and fidelity. Therefore, the proposed novel tools not only complete and improve the existing experimental methodology of HRV-biofeedback, but reach beyond this field into the realm of behavioral interventions.

## Figures and Tables

**Table 1:** Group average (M) with standard deviation (sd) of YETI_AV_, YETI_10_, YETI_20,_ and ΔYETI for both conditions (real and sham training) and results of a Mann-Whitney test between-group comparison (U- and *p*-value) calculated for the original sample and for the per protocol subsample.

	Original Sample				Per Protocol Subsample			
	M_real_ (sd)	M_sham_ (sd)	U	*p*	M_real_ (sd)	M_sham_ (sd)	U	*p*
YETI_AV_	58.67 (1.33)	28.6 (11.55)	33.0	<0.001	6.10 (9.20)	24.33 (7.91)	2.0	<0.001
YETI_10_	57.93 (11.54)	28.02 (12.19)	44.0	<0.001	59.41 (1.52)	24.35 (8.38)	7.0	<0.001
YETI_20_	59.17 (11.51)	28.86 (12.09)	37.0	<0.001	6.54 (1.75)	24.45 (7.92)	2.0	<0.001
ΔYETI	1.23 (9.18)	0.84 (8.59)	385.0	0.737	1.12 (9.51)	0.09 (3.13)	279.0	0.689

**Table 2:** Results of mixed-models analysis of variance (ANOVA) effect of interaction between group (true vs. sham HRV-biofeedback) and training (pre-post and pre-mid-post) calculated on (1) the original sample, (2) the functional clusters, and (3) the per protocol subsample for lnSDNN, lnRMSSD, lnTP, lnLF and lnHF (for 2 timepoints df_s_ = (1,55), df_p*p* =_ (1,47); for 3 timepoints df_s_ = (2,110), df_p*p* =_ (2,94)).

HRV Index	Inter-Action Effects	Original Sample				Functional Cluster				Per Protocol Subsample			
		F(df_s_)	*p*	η_p_^2^	pow	F(df_s_)	*p*	η_p_^2^	pow	F(df_pp_)	*p*	η_p_^2^	pow
ln SDNN	pre-post	4.50	0.038 *	0.08	0.55	7.10	0.010 **	0.11	0.74	6.56	0.014 **	0.12	0.71
	pre-mid-post	2.05	0.133	0.04	0.42	3.29	0.041 *	0.06	0.61	2.79	0.066 *	0.06	0.54
ln RMSSD	pre-post	2.79	0.101	0.05	0.38	6.88	0.011 **	0.11	0.73	5.67	0.021 **	0.11	0.65
	pre-mid-post	1.61	0.205	0.03	0.33	3.39	0.037 *	0.06	0.63	2.70	0.072 *	0.05	0.52
ln TP	pre-post	5.64	0.021 *	0.09	0.65	8.01	0.006 **	0.13	0.79	7.75	0.008 **	0.14	0.78
	pre-mid-post	2.76	0.068	0.05	0.54	4.19	0.018 **	0.07	0.73	3.68	0.029 *	0.07	0.66
ln LF	pre-post	2.45	0.123	0.04	0.34	7.78	0.007 **	0.12	0.78	5.74	0.021 **	0.11	0.65
	pre-mid-post	1.70	0.188	0.03	0.35	4.68	0.011 **	0.08	0.78	3.48	0.035 *	0.07	0.64
ln HF	pre-post	4.75	0.034 *	0.08	0.57	5.03	0.029 **	0.08	0.60	6.07	0.017 **	0.11	0.68
	pre-mid-post	2.81	0.065	0.05	0.54	2.74	0.069 *	0.05	0.53	3.14	0.048 *	0.06	0.59

** interaction significant following the Benjamini–Hochberg correction at FDR = 0.05. * interaction significant following the Benjamini–Hochberg correction at FDR = 0.1.

## Data Availability

The data presented in this study are available on request from the corresponding author. The data are not publicly available due to privacy reasons.

## Supplementary Materials

The following are available online at https://www.mdpi.com/article/10.3390/s21113670/s1: HRV-Biofeedback Training Manual; Figure S1: Schematic representation of the semi-random selection of pre-recorded sham sessions; Table S1 Training Expectancy Questionnaire (TEQ); Table S2 Baseline mean (M) and standard deviation (sd) values of logarithmized HRV parameters (SDNN, RMSSD, TP, LF, and HF) collected at pre-test for both true and sham biofeedback trainees and the results of a Mann-Whitney test between-group comparison (U- and *p*-value); Table S3 Main effects of group (true vs. sham HRV-biofeedback) and training (pre-post), and the interaction effect (group*training) in the mixed-models ANOVA calculated on the whole sample, the functional clusters, and the per protocol subsample for lnSDNN, lnRMSSD, lnTP, lnLF and lnHF (dfs = (1,55), dfpp = (1,47)); Table S4 Post-hoc analysis of the main effects of group (true vs. sham HRV-biofeedback) and training (pre-post), and the interaction effect (group*training) in the mixed ANOVA calculated on the whole sample and the per protocol subsample for lnSDNN, lnRMSSD, lnTP, lnLF and lnHF; Table S5 Main effects of group (true vs. sham HRV-biofeedback) and training (pre-mid-post) and the interaction effect (group*training) in the mixed ANOVA calculated on the whole sample, the functional clusters, and the per protocol subsample for lnSDNN, lnRMSSD, lnTP, lnLF and lnHF (for group: dfs = (1,55), dfpp = (1,47), for training and training*group: dfs = (2,110), dfpp = (2,94)); Table S6 Post-hoc analysis of the main effects of group (true vs. sham HRV-biofeedback) and training (pre-mid-post), and the interaction effect (group*training) in the mixed-models analysis of variance (ANOVA) calculated on the whole sample and the per protocol subsample for lnSDNN, lnRMSSD, lnTP, lnLF and lnHF.
