# Supplementary material for: Novel Methodological Tools for Behavioral Interventions: The Case of HRV-Biofeedback. Sham Control and Quantitative Physiology-Based Assessment of Training Quality and Fidelity
Source: Sensors (Basel). 2021 May 25;21(11):3670. doi: 10.3390/s21113670 (PMC8197468; doi:10.3390/s21113670)
Supplement: Supplementary file 1 [file sensors-21-03670-s001.zip › sensors-1209336-supplementary.pdf]

Supplementary Materials

HRV-Biofeedback Training Manual

<u>**BEFORE YOU START MAKE SURE THAT:**</u>

1. The appropriate screen is projected on the Trainee's monitor depending on the type of training (true vs. sham HRV-biofeedback)
2. The Trainee's <u>MONITOR IS OFF</u>
3. The biofeedback equipment is ready
4. (**SHAM**) The sham recording is prepared
5. The chair is in semi-reclining position, lights are dimmed, air-conditioning is on

---

<u>TRAINING INSTRUCTIONS:</u>

--> Before the training the Trainee should be seated in the chair and asked, if they would like some water or to rest for a while (as sometimes they come by bike or rush not to be late to the training)

First Session

We shall start our first session with a brief introduction to the physiological fenomena underlying HRV-biofeedback training.

Breathing affects the heart rate: upon inhalation the pulse speeds up, and upon exhalation it slows down. These breathing-related changes of heart rate are called „respiratory sinus arrhythmia". It influences the reflexes that control the functioning of the autonomic nervous system, which is responsible for regulation of heart activity, blood pressure, breathing, and many other bodily functions. Furthermore, it affects the body's defense mechanisms and physical fitness, as well as experiencing stress, emotional states and intellectual functioning.

The aim of HRV-biofeedback training is manipulation of heart rate variability (hence the abbreviation HRV). During the training we will be measuring your HRV and present you with the on-line biofeedback information. You will be able to use it to change your HRV and therefore influence the reflexes of the autonomic nervous system. The results of scientific studies confirm the effects of changes in HRV on physical, mental and emotional functioning of the body.

Do you have any questions?

Now, we shall attach to your earlobe an ear clip with the plethysmographic sensor. It is completely noninvasive and sharmless.

--> The researcher attaches the ear clip and presents the software to the Trainee.

This is the software that we will use for training. It presents the biofeedback information regarding your pulse (the bottom left box) and HRV (the upper box). Heart rate variability shows in periods of acceleration and deceleration of the heartbeat, which looks like a sinusoid in the plot. Because HRV depends strongly on the breathing, for every person it is possible to determine a specific breathing frequency which will help to achieve the maximum heart rate variability possible. In the plot it is manifested by an increase in the amplitude and regularity of the sinusoid (high hills and deep valleys).

During the training it is important to pay attention to a couple of things:

1. Breathe **in through the nose**, and **out through pursed lips**. Exhalation should be slightly longer than inhalation.
2. **Do not breathe too deeply**, in order to avoid hyperventilation and light-headedness. If you ever feel dizzy, take more shallow breaths; it is crucial that the pace and depth of breathing is easy to achieve and comfortable to you. Please, do not force anything.
3. Try to **breathe using your diaphragm**. You may check your way of breathing by putting one hand on your belly and one on your chest – upon breathing only the hand on your belly should move up and down significantly following the consecutive breaths. Now try to breathe for a short while in this manner, please.
4. **Positive emotions and attitude** are also very helpful during HRV-biofeedback training. For this reason try not to worry or stress out, but rather attempt to evoke pleasant feelings such as joy, love or gratitude. In order to do that you may remind yourself of places or situations where you experienced such emotions.
5. Changes in HRV are presented not only in the graph; another helpful indicator of training success is the coherence index (the bottom right box). It indicates three levels of success: low (red color), medium (blue) and high (green). In order to achieve better training results try to maintain a high level of coherence, that is, a lot of green color.

If the coherence level is dropping try to counteract by changing one of the parameter of your breathing: depth, pace or rhythm. If these small changes do not result in improved HRV, sometimes it is easier and more advantageous to stop for a moment and "reset yourself" and then resume the training. Please, do not stress out if you find it hard to control the variability of your heart at the beginning – acquiring these skills may require a bit longer and depends on several variables. **Since multiple factors of everyday life influences HRV, such as fatigue, stress, mood, or even diet, the training may be more difficult on some days than others and you may not see steady progress. Sometimes you may simply have a worse day and a temporary low is normal.**

Now I will ask you to try to breathe for a while according to the aforementioned rules: slowly, diaphragmatically, not too deep, inhale through the nose, prolonged exhale through pursed lips, with positive attitude – and attempt to improve your coherence level.

Do you have any questions?

--> NOTE: the following instructions are different for true vs. sham HRV-biofeedback training!

(BIOFEEDBACK) Now, once you understand the breathing technique, we shall proceed with the training. If you wish, we may initially use a pacer that will help you to maintain a slow pace of breathing.

--> If that is the wish of the Trainee, the researcher sets the pacer to 6 bpm. If the Trainee did well during the short test trial there is no need to insist on using the pacer.

The goal of the training is to maintain the coherence level as high as possible for as long as possible. In order to achieve that try to make the sinusoid in the plot as regular and smooth as possible and maximize its amplitude.

(SHAM) Now, once you understand the breathing technique, we shall proceed to use the animations available in the program. Depending on the coherence level achieved, the animation changes color to red, blue or green. The coherence index is also presented at the bottom of the screen in the form of colorful lights. The goal of the training is to maintain the coherence level as high as possible for as long as possible.

Do you have any questions?

If you are ready, we may begin the training.

--> *The researcher prepares the training:*

1. *Starts biofeedback equipment*
2. *(**SHAM**) Starts sham recording*
3. *Only then SWITCHES ON THE MONITOR of the Trainee*
4. *Wishes good luck and exits the room*

--> *Following the training the researcher praises the Trainee, asks how they feel, if they were not light-headed, offers water, thanks for participation in the training session and makes an appointment for the next meeting.*

Following sessions

The researcher may answer Trainee's questions, in the true biofeedback group may suggest to switch the pacer off and guide the breathing for a while. In both groups may remind that:

If something goes wrong and the coherence level falls, try to adjust your breathing.

--> *If the Trainee is promoted to a higher training difficulty level we inform them about the increased level, but NOT about the rules of promoting.*

Today we will promote you to a higher difficulty level. The rules of our training remain the same, only you need to try a bit harder to achieve the consecutive levels of coherence. We started with the easiest settings, and now the program will simply be a bit more demanding,

in order to motivate you to keep training. So do not be discouraged if you get a bit less of the green color; it is the breathing that is important.

> **-->** *It is important to ask the Trainee once in a while how they control their breath and forbid COUNTING!*

Training Expectancy Questionnaire (TEQ)

Table S1 Training Expectancy Questionnaire (TEQ)

Question	Type of answer
1. Did the training influence your state of (underline suitable):<br>a. ...health<br>b. ...physical functioning<br>c. ...mental functioning<br>d. ...emotional functioning	Closed
2. How did you feel during the training?	Open; answers analyzed in 8 categories:<br>a. Relaxed, calm, comforted, quiet<br>b. Bored, weary<br>c. Tired<br>d. Sleepy<br>e. Concentrated, focused on training<br>f. Anxious, worried, nervous, frustrated with the training<br>g. Energetic, ready to act<br>h. Good, fine, normal, as usual
3. Did the training meet your expectations?	Likert-type: 1 – not at all; 5 – somewhat; 9 – very much
4. Do you use the acquired technique at home?	Likert-type: 1 – never; 5 – sometimes; 9 – often
5. I knew what the researchers were testing in this experiment	Likert-type: 1 – strongly disagree; 7 – strongly agree
6. I was not sure what the researchers were trying to demonstrate in this experiment	Likert-type: 1 – strongly disagree; 7 – strongly agree
7. I had a good idea about the hypotheses posed in this experiment	Likert-type: 1 – strongly disagree; 7 – strongly agree
8. I was not sure what the researchers were trying to prove in this experiment	Likert-type: 1 – strongly disagree; 7 – strongly agree
9. Try to finish the following sentence: In	Open

my opinion the experiment was investigating…	
10. Did the additional 10 sessions add or change anything with respect to the initial 10 sessions?	Open; answers analyzed as yes/no

Sham HRV-Biofeedback Training

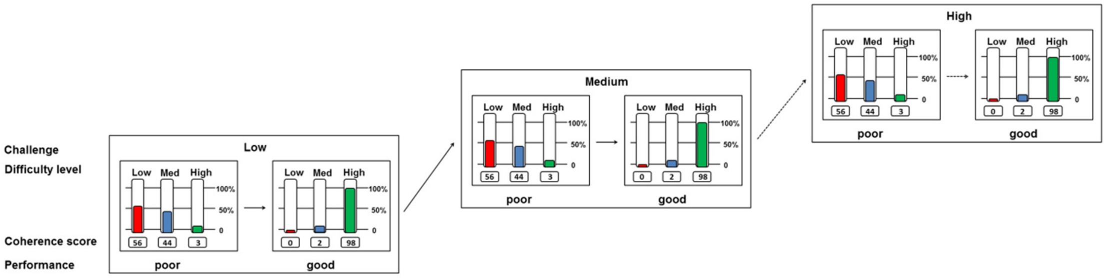

Fig. S1 Schematic representation of the semi-random selection of pre-recorded sham sessions. The recordings were drawn from subsequent sets registered at increasing difficulty levels (‘challenge’). The training started at ‘low’ difficulty and gradually transitioned to ‘medium’ and then ‘high’ (this last step was optional; dashed line). At each difficulty level the sham sessions were randomly chosen at first from a subset characterized by ‘poor’ performance (low coherence values), and later from a subset of ‘good’, successful recordings. This scheme mimics the course of real HRV-biofeedback training, where participants were advanced to a higher difficulty level only after a few sessions with good performance (some trainees never reached ‘high’ difficulty).

Baseline Comparison of HRV Values

Table S2 Baseline mean (M) and standard deviation (sd) values of logarithmised HRV parameters (SDNN, RMSSD, TP, LF and HF) collected at pre-test for both true and sham biofeedback trainees and the results of a Mann-Whitney test between-group comparison (U- and p-value).

	**WHOLE SAMPLE**				**PER PROTOCOL SUBSAMPLE**			
**HRV index**	**M_{true} (sd)**	**M_{sham} (sd)**	**U**	**p**	**M_{true} (sd)**	**M_{sham} (sd)**	**U**	**p**
**lnSDNN**	4.02 (.38)	4.09 (.34)	345	.330	4.01 (.38)	4.16 (.30)	217	.100

**lnRMSSD**	3.53 (.43)	3.70 (.48)	331	.231	3.53 (.44)	3.82 (.43)	205	.060
**lnTP**	7.90 (.82)	8.04 (.69)	357	.434	7.88 (.83)	8.16 (.62)	226	.144
**lnLF**	6.86 (.82)	6.82 (.73)	403	.962	6.81 (.80)	6.95 (.73)	256	.389
**lnHF**	6.20 (.90)	6.21 (1.00)	318	.160	6.21 (.93)	6.77 (.94)	211	.078

ANOVA Results

Table S3 Main effects of group (true vs. sham HRV-biofeedback) and training (pre-post), and the interaction effect (group*training) in the mixed-models ANOVA calculated on the whole sample, the functional clusters, and the per protocol subsample for lnSDNN, lnRMSSD, lnTP, lnLF and lnHF (df_s=(1,55), df_{PP}=(1,47)).

HRV index	Effects	WHOLE SAMPLE				FUNCTIONAL CLUSTER				PER PROTOCOL SUBSAMPLE			
		**F(df$_s$)**	**p**	**η_p^2**	**pow**	**F(df$_s$)**	**p**	**η_p^2**	**pow**	**F(df$_{PP}$)**	**p**	**η_p^2**	**pow**
**ln SDNN**	group	.16	.734	.00	.06	.44	.511	.01	.10	.04	.844	.00	.05
	training	**24.17**	**< .001**	**.31**	**1.00**	**21.35**	**< .001**	**.28**	**1.00**	**18.68**	**< .001**	**.28**	**1.00**
	inter-action	**4.50**	**.038**	**.08**	**.55**	**7.10**	**.010**	**.11**	**.74**	**6.56**	**.014**	**.12**	**.71**
**ln RMSSD**	group	.56	.458	.01	.11	2.73	.104	.05	.37	1.87	.178	.04	.27
	training	**9.40**	**.003**	**.15**	**.85**	**7.82**	**.007**	**.13**	**.79**	**6.32**	**.015**	**.12**	**.69**
	inter-action	2.79	.101	.05	,38	**6.88**	**.011**	**.11**	**.73**	**5.67**	**.021**	**.11**	**.65**
**ln TP**	group	.23	.593	.01	.08	.17	.686	.00	.07	.00	.965	.00	.05
	training	**21.07**	**< .001**	**.28**	**1.00**	**18.10**	**< .001**	**.25**	**.99**	**16.41**	**< .001**	**.26**	**.98**
	inter-action	**5.64**	**.021**	**.09**	**.65**	**8.01**	**.006**	**.13**	**.79**	**7.75**	**.008**	**.14**	**.78**
**ln LF**	group	1.63	.207	.03	.24	.095	.759	.00	.06	.71	.403	.02	.13
	training	**6.56**	**.013**	**.11**	**.71**	**5.23**	**.026**	**.09**	**.61**	3.54	.066	.07	.45
	inter-action	2.45	.123	.04	.34	**7.78**	**.007**	**.12**	**.78**	**5.74**	**.021**	**.11**	**.65**
**ln HF**	group	.46	.501	.01	.10	1.80	.186	.03	.26	1.26	.268	.03	.20
	training	3.43	.070	.06	.44	2.34	.132	.04	.32	2.40	.128	.05	.33
	inter-action	**4.75**	**.034**	**.08**	**.57**	**5.03**	**.029**	**.08**	**.60**	**6.07**	**.017**	**.11**	**.68**

Table S4 Post-hoc analysis of the main effects of group (true vs. sham HRV-biofeedback) and training (pre-post), and the interaction effect (group*training) in the mixed ANOVA calculated on the whole sample and the per protocol subsample for lnSDNN, lnRMSSD, lnTP, lnLF and lnHF.

HRV index	Effects	Samples	WHOLE SAMPLE				PER PROTOCOL SUBSAMPLE			
			μ_1-μ_2	SE	p	95% CI*	μ_1-μ_2	SE	p	95% CI*
ln SDNN	group	true-sham	.03	.09	.733	-.14:.20	-.02	.09	.836	-.20:.16
	training	pre-post	**-.23**	**.05**	**<.001**	**-.33:-.14**	**-.23**	**.05**	**<.001**	**-.34:-.13**
	interaction: group	pre: true-sham	-.07	.10	.474	-.24:.11	-.15	.10	.135	-.34:.40
		post: true-sham	.13	.10	.217	-.07:.33	.11	.11	.323	-.10:.32
	interaction: training	true: pre-post	**-.33**	**.07**	**.002**	**-.47:-.20**	**-.35**	**.08**	**.002**	**-.51:-.20**
		sham: pre-post	**-.13**	**.06**	**.027**	**-.24:-.03**	-.09	.06	.415	-.21:.03
ln RMSSD	group	true-sham	-.15	.10	.140	-.36:.05	**-.24**	**.11**	**.034**	**-.45:-.03**
	training	pre-post	**-.17**	**.06**	**.006**	**-.29:-.06**	**-.16**	**.06**	**.017**	**-.27:-.04**
	interaction: group	pre: true-sham	-.17	.12	.160	-40:0.5	**-.28**	**.12**	**.028**	**-.53:-.05**
		post: true-sham	.02	.11	.868	-.21:.23	-.01	.12	.961	-.24:.22
	interaction: training	true: pre-post	**-.27**	**.07**	**.003**	**-.42:-.12**	**-.29**	**.08**	**.003**	**-.43:-.15**
		sham: pre-post	-.08	.08	.359	-.24:.08	-.01	.09	.932	-.18:.17
ln TP	group	true-sham	.10	.18	.593	-.25:.44	.01	.19	.964	-.36:.38
	training	pre-post	**-.44**	**.10**	**<.001**	**-.64:-.25**	**-.44**	**.11**	**.001**	**-.67:-.22**
	interaction: group	pre: true-sham	-.14	.20	.499	-.52:.26	-.28	.20	.171	-.69:.12
		post: true-sham	.33	.21	.123	-.07:.72	.30	.22	.176	-.12:.72
	interaction: training	true: pre-post	**-.68**	**.15**	**<.001**	**-.98:-.39**	**-.72**	**.16**	**.001**	**-1.03:-.42**
		sham: pre-post	-.22	.12	.093	-.46:.02	-.13	.13	.332	-.39:.11
ln LF	group	true-sham	.25	.19	.209	-.13:.63	.18	.21	.400	-.24:.59
	training	pre-post	**-.32**	**.13**	**.020**	**-.58:-.08**	-.27	.14	.062	-.56:.00
	interaction: group	pre: true-sham	.05	.20	.824	-.34:.44	-.14	.22	.524	-.57:.29
		post: true-sham	.45	.26	.088	-.04:.92	.50	.27	.075	-.04:1.02
	interaction: training	true: pre-post	**-.53**	**.20**	**.020**	**-.94:-.12**	**-.57**	**.22**	**.021**	**-1.00:-.14**
		sham: pre-post	-.13	.15	.421	-.46:-.16	.07	.12	.597	-.18:.32
n H	group	true-sham	-.15	.22	.510	-.62:.29	-.27	.24	.269	-.78:.21

training	pre-post	-.20	.11	.084	-.42:0.02	-.20	.12	.116	-.44:.39
interaction: group	pre: true-sham	-.40	.25	.118	-.88:.07	**-.56**	**.26**	**.042**	**-1.10:-.04**
	post: true-sham	.09	.25	.722	-.41:.56	.02	.26	.957	-.53:.52
interaction: training	true: pre-post	**-.45**	**.16**	**.010**	**-.76:-.13**	**-.47**	**.16**	**.010**	**-.78:-.15**
	sham: pre-post	.04	.15	.814	-.26:.33	.11	.16	.535	-.21:.43

* Calculated using simple bootstrapping with 10000 repetitions

Table S5 Main effects of group (true vs. sham HRV-biofeedback) and training (pre-mid-post) and the interaction effect (group*training) in the mixed ANOVA calculated on the whole sample, the functional clusters, and the per protocol subsample for lnSDNN, lnRMSSD, lnTP, lnLF and lnHF (for group: df_s=(1,55), df_{pp}=(1,47), for training and training*group: df_s=(2,110), df_{pp}=(2,94)).

HRV index	Effects	WHOLE SAMPLE				FUNCTIONAL CLUSTER				PER PROTOCOL SUBSAMPLE			
		$F(df_s)$	p	η_p^2	pow	$F(df_s)$	p	η_p^2	pow	$F(df_{pp})$	p	η_p^2	pow
ln SDNN	group	.10	.749	.00	.06	.69	.410	.01	.13	.09	.761	.00	.06
	training	**12.29**	**< .001**	**.18**	**1.00**	**10.91**	**< .001**	**.17**	**.99**	**9.12**	**< .001**	**.16**	**.97**
	inter-action	2.05	.133	.04	.42	**3.29**	**.041**	**.06**	**.61**	2.79	.066	.06	.54
ln RMSSD	group	1.00	.322	.02	.17	3.36	.072	.06	.44	2.57	.115	.05	.35
	training	**4.89**	**.009**	**.08**	**.80**	**3.98**	**.021**	**.07**	**.70**	3.03	.053	.06	.57
	inter-action	1.61	.205	.03	.33	**3.39**	**.037**	**.06**	**.63**	2.70	.072	.05	.52
ln TP	group	.23	.636	.00	.08	.45	.504	.01	.10	.02	.894	.00	.05
	training	**11.18**	**< .001**	**.17**	**.99**	**9.77**	**< .001**	**.15**	**.98**	**8.34**	**< .001**	**.15**	**.96**
	inter-action	2.76	.068	.05	.54	**4.19**	**.018**	**.07**	**.73**	**3.68**	**.029**	**.07**	**.66**
ln LF	group	1.23	.273	.02	.19	.033	.857	.00	.05	.47	.498	.01	.10
	trainin	**4.64**	**.012**	**.08**	**.77**	**3.80**	**.025**	**.07**	**.68**	2.80	.066	.06	.54

	g												
	inter-action	1.70	.188	.03	.35	**4.68**	**.011**	**.08**	**.78**	**3.48**	**.035**	**.07**	**.64**
**ln HF**	group	1.24	.270	.02	.19	3.21	.079	.06	.42	2.59	.114	.05	.35
	training	1.72	.184	.03	.35	1.31	.275	.02	.28	1.16	.317	.02	.25
	inter-action	2.81	.065	.05	.54	2.74	.069	.05	.53	**3.14**	**.048**	**.06**	**.59**

Table S6 Post-hoc analysis of the main effects of group (true vs. sham HRV-biofeedback) and training (pre-mid-post), and the interaction effect (group*training) in the mixed-models ANOVA calculated on the whole sample and the per protocol subsample for lnSDNN, lnRMSSD, lnTP, lnLF and lnHF.

HRV index	Effects	Samples	WHOLE SAMPLE				PER PROTOCOL SUBSAMPLE			
			μ_1-μ_2	SE	p	95% CI*	μ_1-μ_2	SE	p	95% CI*
ln SDNN	group	true-sham	.03	.09	.750	-.14:.19	-.03	.09	.757	-.21:.15
	training	pre-mid	**-.18**	**.05**	**.001**	**-.29:-.09**	**-.19**	**.06**	**.004**	**-.32:-.07**
		mid-post	-.05	.05	.356	-.14:.05	-.04	.06	.520	-.15:.07
	interaction: group**	mid: true-sham	.02	.11	.831	-.19:.24	-.05	.12	.696	-.28:.19
	interaction: training	true: pre-mid	**-.23**	**.09**	**.021**	**-.43:-.04**	**-.24**	**.10**	**.028**	**-.44:-.05**
		sham: pre-mid	**-.14**	**.05**	**.006**	**-.22:-.06**	**-.14**	**.05**	**.019**	**-.25:-.03**
		true: mid-post	-.10	.09	.284	-.28:.07	-.11	.10	.262	-.30:.08
		sham: mid-post	.01	.04	.908	-.08:.10	.05	.05	.338	-.05:.15
ln RMSSD	group	true-sham	-.10	.10	.319	-.29:.09	-.16	.10	.115	-.36:.03
	training	pre-mid	-.11	.06	.062	-.22:-.00	-.10	.07	.119	.-24:.03
		mid-post	-.06	.05	.293	-.17:.05	-.05	.06	.390	-.17:.06
	interaction: group**	mid: true-sham	-.14	.12	.246	-.37:.09	-.20	.13	.129	-.45:.05
	interaction: training	true: pre-mid	-.13	.09	.191	-.32:.07	-.15	.10	.172	-.35:.06
		sham: pre-mid	-.10	.07	.166	-.22:.03	-.06	.08	.463	-.21:.10
		true: mid-post	-.14	.09	.162	-.33:.04	-.14	.10	.167	-.33:.04
		sham: mid-post	.02	.05	.737	-.09:.13	.05	.06	.382	-.07:.17
ln TP	group	true-sham	.09	.17	.631	-.25:.42	-.03	.18	.891	-.39:.34
	training	pre-mid	**-.34**	**.10**	**.001**	**-.54:-.14**	**-.34**	**.11**	**.003**	**.-.56:-.13**
		mid-post	-.11	.10	.295	-.30:.08	-.10	.11	.386	-.33:.12
	interaction: group**	mid: true-sham	.06	.22	.783	-.38:.51	-.09	.24	.707	-.56:.37
	interaction: training	true: pre-mid	**-.44**	**.17**	**.019**	**-.75:-.12**	**-.43**	**.18**	**.030**	**-.76:-.11**
		sham: pre-mid	**-.24**	**.10**	**.023**	**-.44:-.04**	**-.24**	**.11**	**.043**	**-.45:-.03**
		true: mid-post	-.24	.18	.195	-.59:.10	-.29	.18	.144	-.63:.06
		sham: mid-post	.03	.09	.793	-.15:.20	.11	.10	.301	-.10:.31
n L	group	true-sham	.21	.18	.262	-.15:.56	.14	.20	.495	-.27:.53

	training	pre-mid	**-.27**	**.11**	**.018**	**-.50:-.05**	**-.26**	**.12**	**.032**	**-.49:-.04**
		mid-post	-.05	.11	.638	-.26:.16	-.01	.12	.952	-.26:.23
	interaction: group**	mid: true-sham	.12	.22	.582	-.30:.55	.06	.24	.813	-.42:.52
	interaction: training	true: pre-mid	-.31	.17	.087	-.62:-.00	-.36	.18	.067	-.70:-.03
		sham: pre-mid	-.24	.14	.106	-.55:.05	-.16	.14	.272	-.44:.11
		true: mid-post	-.22	.18	.253	-.59:.15	-.21	.19	.300	-.57:.13
		sham: mid-post	.11	.10	.305	-.08:.31	.23	.11	.060	.00:.45
**HRV index**	**Effects**	**Samples**	**WHOLE SAMPLE**				**PER PROTOCOL SUBSAMPLE**			
			**μ_1-μ_2**	**SE**	**p**	**95% CI***	**μ_1-μ_2**	**SE**	**p**	**95% CI***
**ln HF**	training	pre-mid	-.16	.11	.157	-.37:.06	-.16	.13	.236	-.40:.10
		mid-post	-.04	.13	.759	-.30:.21	-.04	.14	.778	-.33:.24
	interaction: group**	mid: true-sham	-.38	.24	.111	-.87:.08	**-.52**	**.25**	**.046**	**.-1.03:-.03**
	interaction: training	true: pre-mid	-.17	.19	.397	-.53:.20	-.17	.20	.406	-.54:.19
		sham: pre-mid	-.16	.13	.232	-.41:.10	-.14	.15	.385	-.43:.16
		true: mid-post	-.28	.22	.218	-.71:.15	-.29	.22	.211	-.71:.12
		sham: mid-post	.19	.12	.117	-.05:.43	.24	.12	.065	-.00:.47

* Calculated using simple bootstrapping with 10000 repetitions

** Effects of group for interaction at pre- and post- test are identical as in Table A.4
